# Draft genome sequence of *Enterobacter hormaechei* DVZ29, an iodide-oxidizing bacterium isolated from the Hanford site

**DOI:** 10.1128/MRA.00525-23

**Published:** 2023-10-13

**Authors:** Courtney J. Robinson, Robert L. Smith, M. Hope Howard, Danielle L. Saunders, Brady D. Lee

**Affiliations:** 1 Department of Biology, Howard University, Washington, D.C, USA; 2 Pacific Northwest National Laboratory, Energy and Environment Directorate, Richland, Washington, USA; 3 Savannah River National Laboratory, Environmental and Legacy Management Directorate, Aiken, South Carolina, USA; Montana State University, Bozeman, Montana, USA

**Keywords:** *Enterobacter*, bioremediation

## Abstract

*Enterobacter hormaechei* DVZ29 was isolated from a sediment trap incubated in an ^129^I plume at the Hanford Site (Washington State, USA). A whole genome sequencing of the strain resulted in 32 contigs and revealed that the genome is 4.90 Mb, with a G + C content of 55.61%.

## ANNOUNCEMENT

From 1943 to 1987, plutonium was produced at the Hanford Site (Washington State, USA) resulting in the generation of more than 1,000,000,000 m^3^ of liquid wastes, including radioactive materials, which have contaminated the local ecosystems (1). Isolate DVZ29 was among several bacteria we cultured from a sediment trap incubated for 50 days in a groundwater monitoring well located within a plume containing the radioisotope ^129^I at the Hanford Site (2). Its identity was determined using full-length 16S rRNA gene sequencing with the closest match being *Enterobacter hormaechei* strain 0992–77 (NR_042154.1) (2). *E. hormaechei* DVZ29 was identified as an iodide oxidizer, though less efficient than most other bacteria tested, in a study to explore the potential for native bacteria in the soil ecosystem to contribute to bioremediation efforts at the site (2). Here, we report the draft genome of *E. hormaechei* DVZ29 to contribute to the understanding of the genetics of bacterial iodine transformation and bioremediation efforts at the Hanford Site.

*E. hormaechei* DVZ29 was grown from a −80°C freezer stock in tryptic soy broth with shaking for 48 h at 28°C before DNA extraction with the Invitrogen PureLink Genomic DNA Mini Kit. DNA sequencing was performed at SeqCenter (https://www.seqcenter.com). Briefly, the Illumina DNA Prep kit and IDT 10 bp UDI indices were used to generate sample libraries, which were then sequenced on an Illumina NextSeq 2000, resulting in 2 × 151 bp paired-end reads. Demultiplexing, quality control, and adapter trimming were performed with bcl-convert v3.9.3 (https://emea.support.illumina.com/sequencing/sequencing_software/bcl-convert.html). The Bacterial and Viral Bioinformatics Resource Center (BV-BRC; https://www.bv-brc.org/) genome assembly pipeline using Unicycler v0.4.8 assembled reads into contigs (3, 4). CheckM was used to determine the completeness and contamination of the genome, which was then annotated using the Prokaryotic Genome Annotation Pipeline v.6.5 from the National Center for Biotechnology Information (5, 6). In addition to the NCBI average nucleotide identity (ANI) process at genome submission to confirm the identity of DVZ29, the genome sequence data were also uploaded to the Type (Strain) Genome Server (TYGS; https://tygs.dsmz.de) for digital DNA-DNA hybridization (dDDH) analysis (7, 8). Default parameters were used except where otherwise noted.

The *E. hormaechei* DVZ29 genome is 4,903,890 bp in length, with a G + C content of 55.61%, 69 tRNAs, and 4 rRNAs. CheckM analysis indicated that the genome was 99.73% complete, with 0.16% contamination. See Table 1 for additional assembly and sequencing information. The species designation was confirmed with a dDDH (formula d_4_) value of 91.3% (95% CI, 89.2–93.1) and an ANI of >99% when compared to *Enterobacter hormaechei* subsp. *steigerwaltii* DSM 16691 NZ_CP017179.

**TABLE 1:** Information for *E. hormaechei* DVZ29

Parameters	Genome data
Bioproject	PRJNA855226
SRA (Sequence Read Archive) accession	SRR23366688
Genome assembly accession	GCA_030238495.1
Total no. of reads	10,204,820
Total read length (bp)	2,630,013,875
Avg read length (bp)	128
N50 (bp)	511,208
Genome size (bp)	4,903,890
No. of contigs	32
Mean coverage (x)	531
% GC content	55.61
No. of protein coding sequences	4,627
No. of tRNAs	69
No. of rRNAs	4

## Data Availability

This Whole Genome Shotgun project has been deposited in GenBank under the accession number JASSUV000000000. The version described in this paper is JASSUV000000000.1. The raw reads are available under SRA accession number SRR23366688. Additional information is available in Table 1.
